# Thai plants with high antioxidant levels, free radical scavenging activity, anti-tyrosinase and anti-collagenase activity

**DOI:** 10.1186/s12906-017-1994-7

**Published:** 2017-11-09

**Authors:** Moragot Chatatikun, Anchalee Chiabchalard

**Affiliations:** 1 Program in Clinical Biochemistry and Molecular Medicine, Department of Clinical Chemistry, Faculty of Allied Health Sciences, Chulalongkorn University, Bangkok, Thailand; 2 Department of Clinical Chemistry, Faculty of Allied Health Sciences, Chulalongkorn University, Bangkok, 10330 Thailand

**Keywords:** *Ardisia elliptica* Thunb., Antioxidant content, Scavenging activity, Anti-tyrosinase activity, Anti-collagenase activity

## Abstract

**Background:**

Ultraviolet radiation from sunlight induces overproduction of reactive oxygen species (ROS) resulting in skin photoaging and hyperpigmentation disorders. Novel whitening and anti-wrinkle compounds from natural products have recently become of increasing interest. The purpose of this study was to find products that reduce ROS in 14 Thai plant extracts.

**Methods:**

To determine total phenolic and flavonoid content, antioxidant activity, anti-tyrosinase activity and anti-collagenase activity, we compared extracts of 14 Thai plants prepared using different solvents (petroleum ether, dichloromethane and ethanol). Antioxidant activities were determined by DPPH and ABTS assays.

**Results:**

Total phenolic content of the 14 Thai plants extracts was found at the highest levels in ethanol followed by dichloromethane and petroleum ether extracts, respectively, while flavonoid content was normally found in the dichloromethane fraction. Scavenging activity ranged from 7 to 99% scavenging as assessed by DPPH and ABTS assays. The ethanol leaf extract of *Ardisia elliptica* Thunb. had the highest phenolic content, antioxidant activity and collagenase inhibition, while *Cassia alata* (L.) Roxb. extract had the richest flavonoid content. Interestingly, three plants extracts, which were the ethanolic fractions of *Annona squamosa* L., *Ardisia elliptica* Thunb. and *Senna alata* (L.) Roxb., had high antioxidant content and activity, and significantly inhibited both tyrosinase and collagenase.

**Conclusion:**

Our finding show that the ethanol fractions of *Annona squamosa* L., *Ardisia elliptica* Thunb. and *Senna alata* (L.) Roxb. show promise as potential ingredients for cosmetic products such as anti-wrinkle agents and skin whitening products.

## Background

Ultraviolet radiation (UVR) from sunlight is the most significant risk factor for nonmelanoma and melanoma skin cancers [1]. Overexposure to sunlight, in particular UVA and UVB, induces the overexpression of reactive oxygen species (ROS) which damage lipids, proteins and deoxyribonucleic acids. Collagen is the major foundation of the extracellular matrix in the dermis layer of the skin. Excessive ROS increases expression of collagenase, a protease that degrades collagen which can result in photoaging and wrinkling of the skin [2]. In addition, UV exposure induces melanin production resulting in hyperpigmentation. Tyrosinase is the key enzyme initiating skin pigmentation. Firstly, L-tyrosine is hydroxylated to form 3,4-dihidroxyphenylalanine (L-DOPA) by tyrosinase. Subsequently, L-DOPA is oxidized to DOPA quinone by tyrosinase. DOPA quinone is further converted to DOPA chrome that can be converted to 5,6-dihydroxyindole (DHI) or 5,6-dihydroxyindole-2-carboxylic acid (DHICA) [3]. The current treatments for skin aging involves hydroxyl acid to peel the epidermal layer, retinoids to reduce rough skin, and skin filler administered by injecting collagen into the skin. However, these treatments have adverse effects, such as hyperpigmentation, inflammation, cytotoxicity, irritation and bacterial infection [4]. The most popular skin whitening agent is hydroquinone, which inhibits tyrosinase, but its side effects include dermatitis, edema, allergic reactions and ochronosis [5]. Recently, researchers have focused on natural products that inhibit UV-induced ROS, suppress enzymes, and reduce melanin formation as alternatives to current treatments. For example, active phytocompounds, such as arbutin, aloesin, gentisic acid, flavonoids, hesperidin, licorice, niacinamide, yeast derivatives, and polyphenols, inhibit melanogenesis without cytotoxicity to melanocytes [6]. Thus, plants may reduce wrinkle formation and hyperpigmentation caused by sunlight exposure.

The aim of this study was to analyze 14 Thai plants extracted with three different solvents for their potential as anti-wrinkle and skin whitening ingredients. The quantity of antioxidant phenols and flavonoids was evaluated for a correlation with free radical scavenging activities, and anti-collagenase and anti-tyrosinase activities. The extracts had antioxidants that scavenged free radicals and inhibited enzymes involved in wrinkle and pigment formation. We identify *Ardisia elliptica* Thunb., *Annona squamosa* L. and *Senna alata* (L.) Roxb as very promising candidates for use in cosmetic products.

## Methods

### Chemicals and reagents

Folin Ciocalteu’s phenol reagent, sodium carbonate (Na_2_CO_3_), gallic acid, quercetin, 10% aluminium chloride, ethanol, 2, 2-diphenyl-1-picrylhydrazyl (DPPH), ascorbic acid, 2,2^′^-Azino-bis(3-ethylbenzthiazoline-6-sulphonic acid) (ABTS), potassium persulfate, kojic acid, mushroom tyrosinase (EC 1.14.18.1), 3,4-dihydroxy-L-phenylalanine (L-DOPA), N-[3-(2-furyl) acryloyl]-Leu-Gly-Pro-Ala (FALGPA), collagenase from *Clostridium histolyticum* (EC 3.4.24.3), epigallocatechin gallate (EGCG), sodium chloride, calcium chloride and dimethyl sulfoxide (DMSO) were purchased from Sigma-Aldrich Chemical Co. (St. Louis, MO, USA). Petroleum ether, dichloromethane, absolute ethanol, methanol, disodium hydrogen phosphate and sodium dihydrogen phosphate were purchased from Merck (Darmstadt, Germany). All chemicals and reagents were analytical grade.

### Plant materials and extraction

Thirteen species of Thai leaves were collected from the HRH Princess Sirindhorn Herb Garden, Rayong province, Thailand. Mangosteens were obtained from Chanthaburi province, Thailand. These plants were authenticated and deposited at the Herbarium, Department of Botany, Faculty of Science, Chulalongkorn University, Thailand. The scientific names, voucher numbers and plant parts are shown in Table 1. The plants were extracted by using the Soxhlet apparatus. In brief, 10 g of dried plant was extracted separately with petroleum ether, dichloromethane and ethanol. Solvents were removed using a vacuum rotary evaporator under reduced pressure using the MiVac Quattro concentrator. Concentrated samples were dissolved in DMSO at 100 mg/ml and stored at -20 °C until used. Yields of dry extracts are presented in Table 1 as % *w*/w dry plant materials.

**Table 1:** Extracts of 14 Thai plants and their yields based on weight

Voucher number	Parts used	Scientific name	Yield % (*w*/w)		
			Petroleum ether	Dichloromethane	Ethanol
A 015122 (BCU)	Leaf	*Ardisia elliptica* Thunb.	19.89	3.25	31.11
A 015123 (BCU)	Leaf	*Stemona curtisii* Hook.f.	7.55	4.10	6.34
A 015124 (BCU)	Leaf	*Gynura pseudochina* (L.) DC.	8.00	2.76	3.79
A 015125 (BCU)	Leaf	*Senna alata* (L.) Roxb.	5.84	3.52	7.63
A 015126 (BCU)	Leaf	*Croton roxburghii* N.P.Balakr	7.50	4.82	8.17
A 015127 (BCU)	Leaf	*Croton sublyratus* Kurz	7.33	4.03	3.32
A 015128 (BCU)	Leaf	*Phyllanthus acidus* (L.) Skeels	9.70	2.86	4.20
A 015129 (BCU)	Leaf	*Rhinacanthus nasutus* (L.) Kurz	4.43	2.86	5.35
A 015130 (BCU)	Leaf	*Hibiscus mutabilis* L.	6.30	2.79	0.73
A 015131 (BCU)	Leaf	*Streblus asper* Lour.	3.87	2.53	3.56
A 015132 (BCU)	Leaf	*Annona squamosa* L.	8.69	3.81	5.47
A 015133 (BCU)	Leaf	*Datura metel* L.	6.44	4.13	14.15
A 015250 (BCU)	Leaf	*Ipomoea pes-caprae* (L.) R.br.	6.38	4.50	3.98
A 015279 (BCU)	Pericarp	*Garcinia mangostana* Linn.	4.94	11.07	18.64

### Determination of total phenolic content

Total phenolic content of plant extracts was evaluated using the Folin-Ciocalteu method [7]. Briefly, 50 μl of extracts at 1 mg/ml in distilled water was mixed with 50 μl of 10% Folin-Ciocalteu reagent and 50 μl of 0.1 M Na_2_CO_3_. The reaction mixture was incubated for 1 h at room temperature in the dark. Absorbance at 750 nm was measured with a microplate reader (Biotek, USA.). Gallic acid from 1.56 to 100 μg/ml was used as the standard. Total phenolic content of the extracts is expressed as mg gallic acid equivalents (GAE) per g dry plant material. All samples were analyzed in triplicate.

### Flavonoid content determination

Total flavonoid content (TFC) was determined using the aluminium chloride (AlCl_3_) colorimetric assay [7]. Briefly, 50 μl of the extracts at 1 mg/ml in 80% ethanol was mixed with 50 μl of 2% AlCl_3_ solution in the well of a 96 well-plate. The plate was incubated for 15 min at room temperature. The absorbance at 435 nm was measured using a microplate reader. Quercetin from 1.56 to 100 μg/ml served as a standard. Total flavonoid content is expressed as mg quercetin equivalents (QE) per g dry plant material. Samples were analyzed in triplicate.

### DPPH scavenging activity

DPPH scavenging activity assay was performed as described by Yamasaki et al. [8]. DPPH solution was freshly prepared for each assay. Briefly, 100 μg/ml extracts or 1.56 to 100 μg/ml ascorbic acid standard in absolute methanol was mixed with 180 μl of DPPH reagent in a 96 well-plate. The reaction mixture was incubated for 30 min at room temperature in the dark. Then, the absorbance at 517 nm was measured with a microplate reader. The experiments were undertaken in triplicate. The absorbance at 517 nm of DPPH was 0.70 ± 0.02, and decreased absorbance measured scavenging activity. The scavenging ability was calculated as scavenging activity (%) = 100% × [(훥A_517_ of control - 훥A_517_ of sample)/ 훥A_517_ of control]. Percentages of DPPH scavenging activity of the extracts were compared with those of ascorbic acid, and are expressed as mg vitamin C equivalent antioxidant capacity (VCEAC) per g dry plant material. IC50 was determined from a graph of percent inhibition against concentration (from 0.78–100 μg/ml of each extract).

### ABTS scavenging activity

ABTS free radical scavenging activity was performed as previously described [9]. The ABTS^•+^ working reagent was prepared by mixing 7 mM ABTS^•^ and 2.45 mM potassium persulfate at 8:12 volume/volume ratio. The working solution was kept for 16 to 18 h at room temperature in the dark. The ABTS^•+^ solution was diluted with absolute ethanol to give an absorbance at 734 nm of 0.70 ± 0.02. Then, 100 μg/ml extracts or 1.56 to 100 μg/ml ascorbic acid standard in absolute ethanol was added to 180 μl of ABTS^•+^ working reagent in the wells of a 96 well plate. The plate was incubated for 45 min at room temperature, and absorbance was measured at 734 nm. Experiments were undertaken in triplicate. The scavenging ability was calculated as scavenging activity (%) = 100 × [(훥A_734_ of control - 훥A_734_ of sample)/ 훥A_734_ of control]. The percentages of ABTS scavenging activity of the extracts were compared with those of ascorbic acid, and are presented as mg vitamin C equivalent antioxidant capacity (VCEAC) per g dry plant material. IC50 was determined from a graph of percent inhibition against concentration (from 15.62–1000 μg/ml of each extract).

### Determination of mushroom tyrosinase inhibition

The dopachrome method was performed with slight modification [10]. Briefly, 20 μl of plant extracts or DMSO (as control), 20 μl of 203.3 units/ml mushroom tyrosinase and 140 μl of 20 mM phosphate buffer at pH 6.8 were pre-incubated for 10 min at 25 °C. After pre-incubation, 20 μl of 2.5 mM L-DOPA was added and samples were then incubated for an additional 20 min at 25 °C. The amount of dopachrome was measured at 492 nm with a microplate reader. Kojic acid (KA) served as a positive control for inhibition. The percent inhibition of tyrosinase activity (%) was express as % tyrosinase inhibition = 100 × [(훥A_492_ of control – 훥A_492_ of sample)/ 훥A_492_ of control]. The final concentrations of the extracts and kojic acid were 1 and 0.1 mg/ml, respectively. IC50 was determined from a graph of percent tyrosinase inhibition against concentration (from 15.62–1000 μg/ml of each extract).

### Determination of collagenase inhibition

Collagenase inhibition was determined by a previously described method [11]. Briefly, 40 μl of collagenase from *Clostridium histolyticum* at 0.25 units/ml in 50 mM Tricine buffer containing 10 mM CaCl_2_ and 400 mM NaCl, and 10 μl of 50 mM Tricine buffer were mixed with 10 μl of the extracts or DMSO (as control). Epigallocatechin gallate (EGCG) was used as a positive control. After a 15-min incubation at room temperature, 50 μl of N-[3-(2-furyl)acryloyl]-Leu-Gly-Pro-Ala (FALGPA) was added. The absorbance was measured at 340 nm immediately and continually for 20 min. Enzyme activity was evaluated by decreased absorbance during the time interval. The percent inhibition of collagenase activity was calculated as 100 × [(Activity of control – Activity of sample)/ Activity of control]. Final concentrations of the extracts and epigallocatechin gallate were 1 and 0.1 mg/ml, respectively. IC50 was determined from a graph of percent collagenase inhibition against concentration (from 15.62–1000 μg/ml of each extract).

### Statistical analyses

All experiments were carried out in triplicate and results are expressed as mean ± standard error. The correlation coefficiency (R^2^) between antioxidant contents and antioxidant activities was determined by using SigmaPlot version 12.2 software. Difference between two means was evaluated using Student’s *t*-test. Differences were considered significant when the *P*-value was less than 0.05.

## Results

### Extraction yields

Table 1 shows the scientific names, voucher numbers and plant parts of the 14 Thai plants used in this study. The percent yields of the extracts ranged from 0.73% to 31.11% by weight (Table 1). *Ardisia elliptica* Thunb. had the highest yield in the petroleum ether (19.89%) and ethanol extracts (31.11%), whereas *Garcinia mangostana* L. had the highest percent yield from dichloromethane extraction (11.07%).

### Phenolic content of 14 Thai plants

Therefore, total phenolic content in the plants was determined by the Folin-Ciocalteu method. The extracts had a wide range in the quantity of phenols as shown in Table 2, and values varied by 33-fold among the extracts. *Ardisia elliptica* Thunb. had the highest phenol content in all three types of extracts, whereas the lowest phenolic content was present in the *Stemona curtissi* Hook.f. petroleum ether extract.

**Table 2:** Total phenolic and flavonoid contents of 14 Thai plants obtained from different solvents

Extract	Total phenolic content (mg GAE/g dry material)			Total flavonoid content (mg QE/g dry material)		
	Petroleum ether	Dichloromethane	Ethanol	Petroleum ether	Dichloromethane	Ethanol
*Annona squamosa* L.	4.13 ± 0.38	9.26 ± 0.29	62.67 ± 2.32	8.91 ± 0.77	9.70 ± 0.24	12.99 ± 0.65
*Ardisia elliptica* Thunb	22.26 ± 1.77	59.97 ± 2.90	84.00 ± 6.23	19.87 ± 1.26	23.14 ± 1.10	18.56 ± 1.45
*Croton roxburghii* N.P.Balakr	3.57 ± 0.25	9.60 ± 0.46	19.41 ± 0.81	4.25 ± 0.35	12.34 ± 0.29	7.54 ± 0.35
*Croton sublyratus* Kurz	4.73 ± 0.38	6.74 ± 0.51	16.28 ± 0.29	18.55 ± 0.53	20.78 ± 1.49	14.86 ± 0.95
*Datura metel* L.	8.30 ± 0.29	11.43 ± 0.17	18.92 ± 1.50	17.65 ± 1.62	16.77 ± 1.30	2.04 ± 0.16
*Garcinia mangostana* Linn.	19.75 ± 1.44	31.07 ± 2.30	80.79 ± 2.94	5.35 ± 0.10	11.13 ± 0.37	3.20 ± 0.05
*Gynura pseudochina* (L.) DC.	3.26 ± 0.11	9.18 ± 0.65	12.76 ± 0.81	4.87 ± 0.35	18.60 ± 1.06	3.69 ± 0.21
*Hibiscus mutabilis* L.	3.44 ± 0.14	7.14 ± 0.48	17.05 ± 0.64	3.71 ± 0.24	18.79 ± 1.78	3.71 ± 0.09
*Ipomoea pes-caprae* (L.) R.br.	4.72 ± 0.29	11.18 ± 0.53	37.91 ± 3.36	27.48 ± 2.59	18.68 ± 0.66	17.66 ± 0.29
*Phyllanthus acidus* (L.) Skeels	4.65 ± 0.46	10.05 ± 0.74	50.52 ± 2.66	NA	15.80 ± 1.04	11.74 ± 0.74
*Rhinacanthus nasutus* (L.) Kurz	5.04 ± 0.30	9.14 ± 0.39	17.09 ± 1.44	16.98 ± 0.40	19.88 ± 1.98	9.53 ± 0.26
*Senna alata* (L.) Roxb.	4.59 ± 0.21	9.48 ± 0.44	36.83 ± 2.30	7.71 ± 0.36	13.97 ± 1.10	31.38 ± 0.81
*Stemona curtisii* Hook.f.	2.51 ± 0.22	7.76 ± 0.30	59.67 ± 3.28	NA	NA	14.50 ± 0.86
*Streblus asper* Lour.	4.19 ± 0.30	8.22 ± 0.39	23.10 ± 1.84	NA	18.66 ± 1.28	11.29 ± 1.04

Each value is mean ± S.D. of triplicate independent analyses. *GAE* Gallic Acid Equivalent, *QE* Quercitin equivalent, *NA* Not Available

### Flavonoid content of 14 Thai plants

**Fig. 1:**
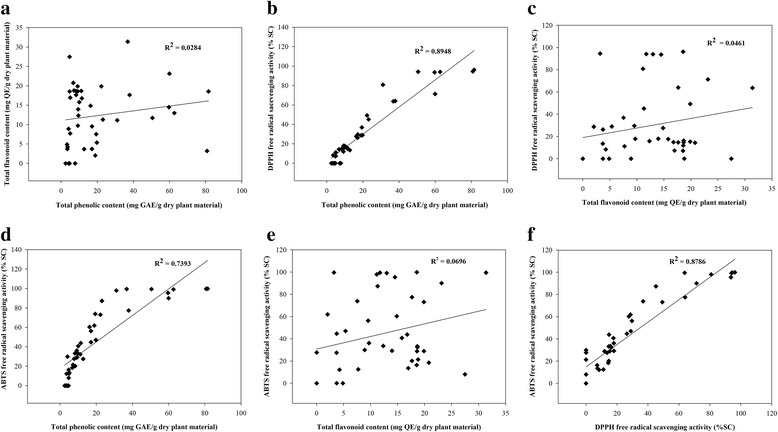
Correlation Analyses. Values in Tables 2 through 4 were evaluated by linear regression analysis, and correlation coefficients expressed as R^2^ are shown in the panels. **a** Total flavonoid content in mg QE/g dry material versus Total phenolic content in mg GAE/g dry material. **b** DPPH percent scavenging activity versus Total phenolic content. **c** DPPH percent scavenging activity versus Total flavonoid content. **d** ABTS percent scavenging activity versus Total phenolic content. **e** ABTS percent scavenging activity versus and Total flavonoid content. **f** ABTS percent scavenging activity versus DPPH percent scavenging activity

Similar to phenols, total flavonoid content varied substantially among the plant species, ranging from 2.04 ± 0.16 to 31.38 ± 0.81 mg QE per g dry material (Table 2). In general, dichloromethane extraction yielded the highest flavonoid level compared with the other solvents. Of all extracts, the highest flavonoid quantity was found in the ethanol extract from *Senna alata* (L.) Roxb leaves (31.38 ± 0.81 mg QE per g dry material). On the other hand, *Ardisia elliptica* Thunb. (23.14 ± 1.10 mg QE per g dry material). had the richest flavonoid content in the dichloromethane fraction. Moreover, *Ipomoea pes-caprae* (L.) R.br. had the highest flavonoid content among the petroleum ether extracts (27.48 ± 2.59 mg QE per g dry material). The lowest detectable flavonoid level was in the ethanol extract from *Datura metel* L. By stark contrast, flavonoids were not found in the petroleum ether and dichloromethane extracts from *Stemona curtisii* Hook.f., and petroleum ether extracts from *Streblus asper* Lour. and *Phyllanthus acidus* (L.) Skeels. Total flavonoid content did not correlate with total phenolic content (R^2^ = 0.0284, Fig. 1a).

### DPPH radical scavenging activity in different extracts from 14 Thai plants

Free radical scavenging activity using DPPH as the indicator is a basic antioxidant assay [12]. As shown in Table 3, scavenging activities of the extracts varied greatly, ranging from 7.11 ± 0.59% to 96.17 ± 0.05%. The *Ardisia elliptica* Thunb ethanol extract had the highest scavenging activity at 96%. Moreover, the next strongest antioxidant activities (> 90%) were observed in ethanol fractions from *Stemona curtisii* Hook.f., *Annona squamosa* L., *Phyllanthus acidus* (L.) Skeels. and *Garcinia mangostana* Linn. In terms of the other solvents, *Ardisia elliptica* Thunb, also had the richest scavenging activity among the petroleum ether fractions, and *Garcinia mangostana* L. had the highest antioxidant activity in the dichloromethane fractions. The lowest scavenging ability was detected in *Croton sublyratus* Kurz in the petroleum ether fraction. No scavenging activity was detected in 7 petroleum ether extracts, and 2 dichloromethane extracts.

**Table 3:** Free Radical Scavenging activity by DPPH assay

Extract	Percent Scavenging Activity (%)			mg VCEAC/g dry weight		
	Petroleum ether	Dichloromethane	Ethanol	Petroleum ether	Dichloromethane	Ethanol
*Annona squamosa* L.	NA	17.91 ± 0.88	94.01 ± 0.40	NA	3.08 ± 0.29	23.60 ± 0.62
*Ardisia elliptica* Thunb.	49.29 ± 1.29	71.35 ± 6.11	96.17 ± 0.05	12.09 ± 0.94	20.23 ± 0.99	24.93 ± 0.19
*Croton roxburghii* N.P.Balakr	8.34 ± 0.57	15.79 ± 1.21	36.89 ± 1.37	1.03 ± 0.09	2.38 ± 0.13	8.45 ± 0.84
*Croton sublyratus* Kurz	7.11 ± 0.59	14.34 ± 0.65	27.64 ± 0.91	0.67 ± 0.07	1.85 ± 0.06	5.29 ± 0.30
*Datura metel* L.	14.67 ± 1.10	14.85 ± 0.64	28.72 ± 0.67	2.00 ± 0.12	2.30 ± 0.06	5.83 ± 0.26
*Garcinia mangostana* Linn.	28.90 ± 0.99	80.87 ± 0.47	94.54 ± 0.15	6.18 ± 0.33	20.42 ± 0.20	24.28 ± 0.20
*Gynura pseudochina* (L.) DC.	NA	11.90 ± 0.71	13.54 ± 0.67	NA	1.49 ± 0.03	1.82 ± 0.13
*Hibiscus mutabilis* L.	NA	NA	26.28 ± 0.93	NA	NA	4.29 ± 0.21
*Ipomoea pes-caprae* (L.) R.br.	NA	16.27 ± 0.14	64.06 ± 1.23	NA	2.49 ± 0.19	16.79 ± 0.41
*Phyllanthus acidus* (L.) Skeels	NA	17.64 ± 1.05	94.17 ± 0.61	NA	3.06 ± 0.28	23.84 ± 0.73
*Rhinacanthus nasutus* (L.) Kurz	7.38 ± 0.46	15.35 ± 1.27	29.63 ± 1.41	1.01 ± 0.08	2.32 ± 0.21	6.50 ± 0.16
*Senna alata* (L.) Roxb.	11.18 ± 0.99	17.99 ± 0.61	63.74 ± 0.54	1.48 ± 0.14	3.13 ± 0.31	15.35 ± 0.13
*Stemona curtisii* Hook.f.	NA	NA	93.63 ± 0.22	NA	NA	23.55 ± 0.55
*Streblus asper* Lour.	NA	14.65 ± 0.92	45.14 ± 0.67	NA	1.94 ± 0.13	10.45 ± 0.30

Each value is mean ± S.D. of triplicate independent analyses. Calculations of values are described in the Materials and Methods section. VCEAC is Vitamin C Equivalent Antioxidant capacity, and NA denotes not available

### ABTS radical scavenging activity in different extracts from 14 Thai plants

Antioxidant activity of aqueous and lipid phases in the plants has also been evaluated by a decolorization assay using ABTS [13]. Again, ascorbic acid served as the standard antioxidant. As with the DPPH assay, scavenging activity in the ABTS assay varied greatly among the plant preparations with a similar broad range from 8.03 ± 0.54% to 99.84 ± 0.07% (Table 4). Furthermore, the next strongest scavenging activities (> 90%) were observed in the same 4 ethanol fractions as shown by the DPPH assay. In addition, no scavenging activity was found in the same 5 petroleum ether extracts. In general, the values obtained with the ABTS assay were higher than the DPPH values. Hence, activity in the ethanol extract from *Senna alata* (L.) Roxb. was now observed as >90%, and scavenger activity was detected in all dichloromethane extracts, and petroleum ether extracts from *Annona squamosa* L. and *Ipomoea pes-caprae* (L.) R.br. which was not detected by the DPPH assay.

**Table 4:** Scavenging activity by ABTS assay

Extract	%Scavenging activity (%SC)			mg VCEAC/g dry weight		
	Petroleum ether	Dichloromethane	Ethanol	Petroleum ether	Dichloromethane	Ethanol
*Annona squamosa* L.	29.89 ± 0.79	36.31 ± 0.60	99.13 ± 0.29	25.76 ± 0.76	34.40 ± 0.32	96.15 ± 0.38
*Ardisia elliptica* Thunb.	73.08 ± 1.48	90.01 ± 0.54	99.84 ± 0.07	65.28 ± 1.86	86.34 ± 1.07	96.97 ± 0.34
*Croton roxburghii* N.P.Balakr	12.19 ± 0.91	33.54 ± 0.65	73.86 ± 1.22	9.14 ± 0.45	33.22 ± 0.46	69.62 ± 0.90
*Croton sublyratus* Kurz	16.34 ± 0.69	18.47 ± 0.73	60.36 ± 0.17	13.07 ± 1.29	17.81 ± 1.20	58.62 ± 0.68
*Datura metel* L.	20.17 ± 0.68	43.79 ± 0.89	61.94 ± 0.50	16.42 ± 0.65	42.36 ± 1.11	60.06 ± 0.16
*Garcinia mangostana* Linn.	47.01 ± 2.73	97.96 ± 0.85	99.66 ± 0.05	45.10 ± 2.45	95.19 ± 1.43	96.52 ± 0.34
*Gynura pseudochina* (L.) DC.	NA	28.88 ± 0.45	27.52 ± 1.66	NA	28.00 ± 0.36	25.37 ± 1.99
*Hibiscus mutabilis* L.	NA	21.36 ± 0.51	44.74 ± 0.23	NA	20.34 ± 0.42	43.29 ± 1.26
*Ipomoea pes-caprae* (L.) R.br.	8.03 ± 0.54	32.30 ± 1.93	77.43 ± 1.74	4.53 ± 0.34	31.41 ± 2.73	75.16 ± 2.37
*Phyllanthus acidus* (L.) Skeels	NA	40.72 ± 1.25	99.43 ± 0.17	NA	40.06 ± 1.90	96.27 ± 0.10
*Rhinacanthus nasutus* (L.) Kurz	13.55 ± 0.49	28.90 ± 0.57	56.26 ± 0.90	10.60 ± 0.24	28.29 ± 1.77	53.26 ± 1.95
*Senna alata* (L.) Roxb.	12.51 ± 0.41	29.21 ± 1.35	99.45 ± 0.11	8.79 ± 0.17	28.97 ± 0.56	96.28 ± 0.12
*Stemona curtisii* Hook.f.	NA	27.65 ± 1.30	95.49 ± 0.25	NA	27.48 ± 0.11	91.91 ± 0.63
*Streblus asper* Lour.	NA	33.22 ± 0.77	87.32 ± 0.39	NA	32.92 ± 1.57	83.78 ± 0.90

Each value is mean ± S.D. of triplicate independent analyses. Calculations of values are described in the Materials and Methods section. *VCEAC* Vitamin C Equivalent Antioxidant capacity, *NA* not available

### Tyrosinase activity inhibition by plant extracts

The ability of compounds from the Thai plants to inhibit mushroom tyrosinase activity was evaluated using an in vitro assay with L-DOPA as the substrate. Kojic acid served as a known inhibitor, and caused maximal enzymatic inhibition of 93.38 ± 1.63%. As shown in Table 5, only ethanol extracts significantly inhibited tyrosinase activity, with *Ardisia elliptica* Thunb. preparations being the exception. The petroleum ether and dichloromethane fractions of *Ardisia elliptica* Thunb. inhibited tyrosinase activity by approximately 20%. The ethanol fraction from *Rhinacanthus nasutus* (L.) Kurz (IC 50 value of 271.50 μg/ml) was the most potent tyrosinase inhibitor, followed by the ethanol extracts from *Ardisia elliptica* Thunb. and *Phyllanthus acidus* (L.) Skeels. Other ethanol fractions significantly decreased enzymatic activity by more than 20% (Table 5), whereas the remaining extracts did not have detectable inhibitory activity (data not shown).

**Table 5:** Inhibition of tyrosinase and collagenase activities by Thai plant extracts

Plant	Extract	Tyrosinase inhibition (%)	Collagenase inhibition(%)
*Annona squamosa* Linn.	Ethanol	21.92 ± 1.45^**^	55.12 ± 3.18^***^
*Ardisia elliptica* Thunb.	Petroleum ether	19.42 ± 1.21^**^	NA
*Ardisia elliptica* Thunb.	Dichloromethane	21.40 ± 1.61^**^	NA
*Ardisia elliptica* Thunb.	Ethanol	49.54 ± 1.23^***^	94.88 ± 6.93^***^
*Croton sublyratus* Kurz	Ethanol	NA	24.14 ± 0.98^**^
*Datura metel* L.	Ethanol	20.91 ± 1.70^**^	NA
*Ipomoea pes-caprae* (L.) R.br.	Ethanol	23.01 ± 1.65^**^	NA
*Phyllanthus acidus* (L.) Skeels.	Ethanol	42.92 ± 3.85^***^	NA
*Rhinacanthus nasutus* (L.) Kurz	Ethanol	64.68 ± 5.46^***^	NA
*Senna alata* (L.) Roxb.	Ethanol	23.49 ± 1.09^**^	41.49 ± 2.63^***^
Kojid acid (tyrosinase inhibitor)		93.38 ± 1.63^***^	
Epigallatecathechin gallate (collagenase inhibitor)		–	90.51 ± 2.79^***^

Each value is mean ± S.D. of triplicate independent analyses. Significantly different from the control group

(^*^ *P* < 0.05, ^**^ *P* < 0.01, ^***^ *P* < 0.001). *NA* not available

### Collagenase activity inhibition by 14 plants

Extracts were tested for anti-collagenase activity using *Clostridium histolyticum* collagenase, and N-[3-(2-furyl)acryloyl]-Leu-Gly-Pro-Ala (FALGPA) as the substrate. Epigallatecatechin gallate is a known collagenase inhibitor, and decreased enzymatic activity by 90.51 ± 2.79%. As shown in Table 5, only 4 ethanol extracts contained detectable collagenase inhibitory activity. Of those causing inhibition, *Ardisia elliptica* Thunb. (IC 50 value of 157.78 μg/ml) exhibited the highest level of collagenase inhibition, followed by *Annona squamosa* L. (IC 50 value of 426.67 μg/ml), *Senna alata* (L.) Roxb., and *Croton sublyratus* Kurz in rank order. Other plant extracts did not significantly inhibit collagenase activity under the reaction conditions utilized in this study (data not shown).

## Discussion

Solar radiation is a significant environmental factor in skin damage and can induce skin cancer [14]. UV radiation causes a pro-inflammatory response, extracellular matrix degradation and antioxidant depletion [15, 16]. UV causes formation of reactive oxygen species (ROS) that induce hyperpigmentation and collagenase expression [17, 18]. Our study investigated 14 Thai plants extracted with three different solvents for their potential as anti-wrinkle and skin whitening ingredients. In this study, we used petroleum ether, dichloromethane and ethanol for plant extraction using Soxhlet apparatus. *Ardisia elliptica* Thunb. had the highest yield in the petroleum ether and ethanol extracts, whereas *Garcinia mangostana* L. had the highest percent yield from dichloromethane extraction. These solvents are a series of organic solvents with increasing polarities. Variation among the percent yields depended on the plant species, and probably reflected differences in chemical composition of the plants.

Phenolics are the largest group of phytochemicals found in plants and they have various biological activities in animals, including humans [19]. Total phenolic content in the plants was determined by the Folin-Ciocalteu method. Overall, the ethanol fraction had the richest phenolic content, followed by dichloromethane, while petroleum ether with low polarity had the lowest phenolic content compared to the other solvents. In this study, *Ardisia elliptica* Thunb. had the highest phenolic content in all three types of extracts. In previous studies, dichloromethane leaf extracts of *Ardisia elliptica* Thunb. have a phenolic content of 101 ± 1.3 mg GAE per g dry plant material, which is more than the content in a twig extract [20]. Moreover, a methanol extract of ripe *Ardisia* fruit contained 5.64 ± 0.37 g GAE per 100 g extract [21]. Hence, leaves and fruits of *Ardisia elliptica* Thunb. have a high phenolic content that can be easily extracted with methanol, dichloromethane and ethanol.

Flavonoids are pigments in flowers, leaves, fruits and seeds. These compounds are secondary metabolites of plants and are widely distributed among plant species [22]. Next, the flavonoid content within the Thai plants was evaluated using the aluminium chloride colorimetric assay. Our results showed that the highest flavonoid quantity was found in the ethanol extract from *Senna alata* (L.) Roxb leaves. In a previous study, high flavonoid content was found in water (4.25 mg QE per 100 g) and methanol fractions (3.97 mg QE per 100 g) of *Senna alata* (L.) Roxb. [23]. Thus, *Senna alata* (L.) Roxb preparations have a high flavonoid content when extracted with high polarity solvents including ethanol, methanol and water. *Ardisia elliptica* Thunb. had the richest flavonoid content in the dichloromethane fraction. Fruit of this plant also has a high flavonoid content 36.91 ± 2.37 mg QE per g extract [24]. Hence, fruit and leaves of *Ardisia elliptica* Thunb. are rich in flavonoids. Total flavonoid content did not correlate with total phenolic content. However, flavonoids have many biological activities such as UVB protection [25], anti inflammatory [26], anti-hepatotoxicity [27] and anti cancer [28].

Free radical scavenging activity using DPPH and ABTS assay. In the DPPH assay, DPPH receives a hydrogen atom from an antioxidant [29]. We found that *Ardisia elliptica* Thunb ethanol extract had the highest scavenging activity. Other investigators have also reported that dichloromethane fractions of *Ardisia elliptica* Thunb. leaves and stems have high levels of antioxidant activity as determined by the DPPH assay, and, hence, this plant is very interesting to investigate further as a herbal treatment [20]. The extracts from the ethanol fraction with high polarity clearly showed better antioxidant activity than fractions with lower polarities containing dichloromethane and petroleum ether. Ethanol extracts contained the highest levels of free radical scavenging activity compared with the other extracts, and all ethanol extracts were active. In the ABTS assay, ABTS is converted to its radical cation by the addition of potassium persulfate. In the presence of an antioxidant, the reactive ABTS cation (or ABTS^•+^) is converted to its colorless natural form [9]. In agreement with the DPPH assay, ethanol extracts contained the highest levels of scavenger activity as compared with the other extracts. Again, the highest scavenging activities in ethanol, dichloromethane and petroleum ether extracts were from the same plants as shown by the DPPH assay. The results of the DPPH and ABTS assays were highly correlated as expected (Fig. 1f).

However, total flavonoid content of the plant extracts did not correlate with free radical scavenger activity as detected by the DPPH assay (Fig. 1c) or by the ABTS assay (Fig. 1e). Our findings of no significant relationship between flavonoid content and scavenger activity using the ABTS assay is consistent with other investigators’ results [30]. By contrast, total phenolic content of the plant preparation positively correlated with scavenger activity measured by both assays (Fig. 1b and d) in agreement with a previous study [31]. Noticeably, the scavenging activity depended on total phenolic content and solvents with high polarity, such as ethanol and dichloromethane. These results suggest that the phenolic content is the major constituent with antioxidant activity in the 14 Thai plants.

Melanin, the major pigment of skin and hair color, is synthesized by melanocytes within melanosomes. Overproduction and accumulation of melanin in skin may lead to pigmentary disorders and aesthetic problems. Hyperpigmentation occurs in sun-exposed areas of the skin [32]. In the melanogenesis, tyrosinase is the key enzyme in the rate-limiting step in which L-tyrosine is hydroxylated to L-DOPA, which is further oxidized into DOPAquinone. After that, it is converted into DOPAchrome that is a substrate for melanin synthesis [3]. Downregulation of tyrosinase activity has been proposed to be responsible for decreased melanin production. The development of novel whitening phytochemical compounds from natural products has recently become a growing trend. Our finding showed that the ethanol fraction from *Rhinacanthus nasutus* (L.) Kurz was the most potent tyrosinase inhibitor, followed by the ethanol extracts from *Ardisia elliptica* Thunb. and *Phyllanthus acidus* (L.) Skeels. Obviously, 7 plants from 14 plants had the high phenolic content, especially *Ardisia elliptica* Thunb. and *Annona squamosa* L.. Moreover, *Senna alata* (L.) Roxb. had the richest flavonoid content which can inhibit tyrosinase activity. Active compounds from the plants such as arbutin, aloesin, gentisic acid, flavonoids, hesperidin, licorice, niacinamide, yeast derivatives, and polyphenols, can inhibit melanogenesis without cytotoxicity to melanocytes [6].

Collagenase is a transmembrane zinc peptidase that cleaves the X-Gly bond of collagen. Collagen is an abundant structural protein and extracellular matrix component [33]. Decreased collagen and elastin fibers increases with age and damage from UV radiation inducing wrinkled skin [34]. Collagenase inhibition has been proposed to prevent skin aging. Of those causing inhibition in our study, *Ardisia elliptica* Thunb. exhibited the highest level of collagenase inhibition, followed by *Annona squamosa* L., *Senna alata* (L.) Roxb., and *Croton sublyratus* Kurz in rank order. In a previous study, cocoa pod extract had phenolic acid and flavonoids that inhibited elastase and collagenase activity [35]. Notably, three ethanol extracts (*Ardisia elliptica* Thunb., *Annona squamosa* L. and *Senna alata* (L.) Roxb. inhibited both tyrosinase and collagenase. These plants also had high phenolic and flavonoid levels, and antioxidant activity. Interestingly, these extracts have possible uses as ingredients for cosmetic products.

## Conclusion

Our results demonstrate that extracts of 14 Thai plants had varying degrees of total phenolic and flavonoid content as well as free radical scavenging activities, depending on the extraction solvents. There was a high correlation between total phenolic content and free radical scavenging activity ass assessed by the DPPH and ABTS assays. The ethanol fraction of *Ardisia elliptica* Thunb. had the highest phenolic content, followed by *Annona squamosa* L. Both plants significantly inhibited tyrosinase and collagenase activities, while *Rhinacanthus nasutus* (L.) Kurz showed the highest tyrosinase inhibition. Moreover, *Senna alata* (L.) Roxb. was richest in flavonoid content, and also exhibited tyrosinase and collagenase inhibitory behavior. The ethanol fraction of three plants, namely *Annona squamosa* L., *Ardisia elliptica* Thunb and *Senna alata* (L.) Roxb., have the potential to be ingredients in cosmetic products for anti-wrinkling as well as skin whitening. Further studies are necessary to investigate the active components and safety of these extracts.

## Abbreviations

ABTS: 2,2^′^-Azino-bis(3-ethylbenzthiazoline-6-sulphonic acid)
DHI: 5,6-dihydroxyindole (DHI)
DHICA: 5,6-dihydroxyindole-2-carboxylic acid
DMSO: Dimethyl sulfoxide
DPPH: 2,2-diphenyl-1-picrylhydrazyl
EGCG: Epigallocatechin gallate
FALGPA: N-[3-(2-furyl) acryloyl]-Leu-Gly-Pro-Ala
GAE: Gallic acid equivalents
KA: Kojic acid
L-DOPA: 3,4-dihidroxyphenyl alanine
Na_2_CO_3_: Sodium carbonate
QE: Quercetin equivalents
ROS: Reactive oxygen species
SC: Scavenging activity
TFC: Total flavonoid content
TPC: Total phenolic content
UVR: Ultraviolet radiation
VCEAC: Vitamin C equivalent antioxidant capacity
